# Optimizing e-Consultations to Adolescent Medicine Specialists: Qualitative Synthesis of Feedback From User-Centered Design

**DOI:** 10.2196/25568

**Published:** 2021-08-05

**Authors:** Jacquelin Rankine, Deepika Yeramosu, Loreta Matheo, Gina M Sequeira, Elizabeth Miller, Kristin N Ray

**Affiliations:** 1 Division of Adolescent and Young Adult Medicine, Department of Pediatrics University of Pittsburgh School of Medicine UPMC Children's Hospital of Pittsburgh Pittsburgh, PA United States; 2 Department of Pediatrics University of Pittsburgh School of Medicine UPMC Children's Hospital of Pittsburgh Pittsburgh, PA United States; 3 Division of Adolescent Medicine Seattle Children's Hospital Seattle, WA United States

**Keywords:** referral, consultation, telemedicine, telehealth, adolescents, child health, child health services, confidentiality, access to health care

## Abstract

**Background:**

e-Consultations between primary care physicians and specialists are a valuable means of improving access to specialty care. Adolescents and young adults (AYAs) face unique challenges in accessing limited adolescent medicine specialty care resources, which contributes to delayed or forgone care. e-Consultations between general pediatricians and adolescent medicine specialists may alleviate these barriers to care. However, the optimal application of this model in adolescent medicine requires careful attention to the nuances of AYA care.

**Objective:**

This study aims to qualitatively analyze feedback obtained during the iterative development of an e-consultation system for communication between general pediatricians and adolescent medicine specialists tailored to the specific health care needs of AYAs.

**Methods:**

We conducted an iterative user-centered design and evaluation process in two phases. In the first phase, we created a static e-consultation prototype and storyboards and evaluated them with target users (general pediatricians and adolescent medicine specialists). In the second phase, we incorporated feedback to develop a functional prototype within the electronic health record and again evaluated this with general pediatricians and adolescent medicine specialists. In each phase, general pediatricians and adolescent medicine specialists provided *think-aloud* feedback during the use of the prototypes and semistructured exit interviews, which was qualitatively analyzed to identify perspectives related to the usefulness and usability of the e-consultation system.

**Results:**

Both general pediatricians (n=12) and adolescent medicine specialists (n=12) perceived the usefulness of e-consultations for AYA patients, with more varied perceptions of potential usefulness for generalist and adolescent medicine clinicians. General pediatricians and adolescent medicine specialists discussed ways to maximize the usability of e-consultations for AYAs, primarily by improving efficiency (eg, reducing documentation, emphasizing critical information, using autopopulated data fields, and balancing specificity and efficiency through text prompts) and reducing the potential for errors (eg, prompting a review of autopopulated data fields, requiring physician contact information, and prompting explicit discussion of patient communication and confidentiality expectations). Through iterative design, patient history documentation was streamlined, whereas documentation of communication and confidentiality expectations were enhanced.

**Conclusions:**

Through an iterative user-centered design process, we identified user perspectives to guide the refinement of an e-consultation system based on general pediatrician and adolescent medicine specialist feedback on usefulness and usability related to the care of AYAs. Qualitative analysis of this feedback revealed both opportunities and risks related to confidentiality, communication, and the use of tailored documentation prompts that should be considered in the development and use of e-consultations with AYAs.

## Introduction

### Background

Although general pediatricians manage a variety of adolescent health concerns, referrals to adolescent medicine specialists are common. Common referrals to adolescent medicine specialists include management of menstrual disorders, sexual and reproductive health care, gender-affirming care, and behavioral health care [1]. However, adolescents and young adults (AYAs) experience barriers to completing these referrals and receiving adolescent medicine specialty care. The scarcity of board-certified adolescent medicine specialists is itself a critical barrier to the care of AYAs. The number of adolescent medicine specialists per state ranges from 3.4 adolescent medicine specialists per 100,000 children in Rhode Island to 0 adolescent medicine specialists in 4 states and Puerto Rico [2]. Owing to this lack of adolescent medicine specialists, travel distance is substantial, and appointment availability does not meet demand [3]. As a result, AYAs and their families face significant travel and time burdens, financial costs, and delays in care when seeking adolescent medicine specialty care [4-6], with these barriers falling disproportionately on families with lower socioeconomic status [7]. Even with the rapidly increasing use of live-interactive telemedicine for adolescent medicine specialty care in the context of the COVID-19 pandemic [8], both patient-side barriers (eg, low health literacy and limited internet access) and system-side barriers (eg, inadequate supply of adolescent medicine specialists to meet referral demand) remain [9].

For AYAs referred to adolescent medicine specialists for confidential health concerns, the barriers to attending these visits without the knowledge or assistance of their families may be increased or insurmountable. Confidentiality concerns have been associated with forgone care [10], decreased receipt of sexual and reproductive health services and contraception [11], and decreased screening for sexually transmitted infections among AYAs [12]. Barriers to completed referrals with pediatric subspecialists may lead primary care physicians (PCPs) to seek clinical guidance from subspecialists through alternative routes such as phone calls, text messaging, emails, or in-person *curbside consultations* [13]. These informal methods of consultation are often not accompanied by compensation for clinicians’ time or the formal documentation necessary for medical-legal purposes and ongoing care management.

e-Consultations are an alternative strategy for subspecialists to offer clinical guidance to PCPs. In this asynchronous model of telehealth care, a PCP submits patient-specific information to a subspecialist, who later reviews this information and provides recommendations back to the PCP to guide their care [14]. In some cases, patient-specific guidance provided by subspecialists through e-consultations may allow PCPs to manage the patient without requiring an in-person subspecialist visit. In other cases, e-consultations may guide PCPs in evaluation or management while awaiting a subspecialist visit. e-Consultations are now used in many health systems, with evidence suggesting that this form of virtual triage and management increases access to subspecialist expertise and timeliness of appointments for patients who still require in-person subspecialty care [15-17].

Although e-consultations have the potential to increase access to adolescent medicine specialists, the application of this model within adolescent medicine requires careful attention to the nuances of adolescent care. Issues of confidentiality, consent, and communication among PCPs, adolescent medicine specialists, AYAs, and caregivers of AYAs warrant specific focus. In addition, consideration of the optimal information to transfer between PCPs and adolescent medicine specialists [18] and details of workflow, training, and documentation are needed to promote high-quality care while maintaining an efficient workflow for both PCPs and adolescent medicine specialists.

### Objectives

In this study, we seek to examine feedback during the iterative development of e-consultations for communication between PCPs and adolescent medicine specialists. Informed by prior studies examining e-consultation features and design [19], we develop an initial prototype and iteratively modify it through cycles of user-centered design. In this manuscript, we share our development process, feedback themes through each design stage, and the documentation templates and workflow developed through this process.

## Methods

### Study Design

We conducted an iterative user-centered design and evaluation process in two phases. In phase 1, we developed a prototype e-consultation template and workflow based on a literature review and conversations with individuals using e-consultations in other settings, including the Veterans Health Administration and University of Pittsburgh Medical Center. We evaluated the static prototype and workflow storyboards with target users, including general pediatrician PCPs and adolescent medicine specialists. We incorporated feedback from this process to develop a functional prototype within the electronic health record (EHR) and again evaluated this with PCPs and adolescent medicine specialists in phase 2. We performed a qualitative analysis of the feedback obtained through *think-aloud* commentary during the use of the prototypes and semistructured exit interviews to identify perspectives regarding e-consultations for AYA care.

### Recruitment

We approached target users via email from an existing research network of primary care practices (Pediatric PittNet) and the Division of Adolescent and Young Adult Medicine of the University of Pittsburgh Medical Center Children’s Hospital of Pittsburgh. The Division of Adolescent and Young Adult Medicine is the primary group of adolescent medicine specialists caring for AYAs across 26 counties in Western Pennsylvania as well as portions of Ohio and West Virginia. The general pediatrician PCPs across more than 40 practices affiliated with the Children’s Hospital of Pittsburgh span both academic and community practices across 13 counties in Western Pennsylvania. Approximately one-quarter of these practices are in rural counties, and all use a shared EHR, which is also used by the Division of Adolescent and Young Adult Medicine. At the time of this study, no payers in our region paid clinicians for their time conducting store-and-forward provider-to-provider consultations. General pediatrician PCPs and adolescent medicine specialists were eligible for participation, including both academic and community general pediatricians, advanced practice providers, and trainees in fellowship. Clinicians who were not engaged in patient care were excluded from the study. The participants received US $25 gift cards. Participants were purposefully sampled to ensure a balance between PCPs and adolescent medicine specialists and diversity of years of experience and clinical effort. The University of Pittsburgh institutional review board provided ethical approval. We obtained written documentation of informed consent from participants.

### Iterative Design Process

#### Phase 1: Static Prototype

Phase 1 was conducted from July to August 2018. A study team member (DY or KNR) met with each participant in a private office, obtained informed consent, and talked through a series of 22 slides. These slides reviewed the goals of e-consultations, the research process, prototype note templates for both PCPs and adolescent medicine specialists, and storyboards that provided a visual representation of planned workflow and prototype screenshots illustrating the planned process as a clinician worked through the system. We started with these static prototypes because they could be developed with minimal time investment and rapidly adapted in response to feedback. During this phase, data were collected through think-alouds, exit interviews, and web-based surveys. Changes were made to the prototype after every 3 participants to continuously incorporate feedback.

#### Phase 2: EHR Prototype

After incorporating feedback from phase 1, we developed a functional electronic prototype within the play environment of our EHR (EpicCare). Phase 2 was conducted from July to September 2019. In this phase, participants were asked to complete specific tasks depending on their role (PCP vs adolescent medicine specialist).

During PCP sessions, participants were given 3 vignettes where adolescent medicine specialist advice might be sought related to polycystic ovary syndrome, gender-affirming care, and eating disorders. Participants were asked to prepare mock e-consultations for each vignette within a practice EHR environment. During the first 2 vignettes, PCPs provided think-aloud commentary while completing the task. For the third vignette, participants completed the notes at their usual working pace while their efforts were timed. For the final 3 PCP participants, mock responses created by prior adolescent medicine specialist participants were shared after the prior session components, and the PCPs were asked to *think aloud* as they reviewed and interpreted this mock advice.

During adolescent medicine specialist sessions, participants were given 3 mock e-consultations (1 for each vignette) generated by PCP participants during prior PCP sessions without identifiers of the participating PCP. During the first 2 vignettes, adolescent medicine specialists provided think-aloud commentary while preparing mock responses. For the third vignette, participants completed the mock response at their usual working pace while their efforts were timed.

After completing these tasks, all participants completed semistructured exit interviews and the same web-based survey used in phase 1. Throughout this phase, changes were again continuously made to the EHR prototype in response to feedback.

### Data Collection and Analysis

Throughout the iterative user-centered design and evaluation process, feedback was collected from PCPs and adolescent medicine specialists through the processes described above: think-aloud commentary during the use of the prototypes and semistructured exit interviews. During think-alouds, participants were asked to talk through their thoughts as they reviewed the prototypes and were encouraged to comment on possible limitations or undesirable components of the proposed system. The semistructured exit interviews included questions on the perceived usefulness and usability of the e-consultation system.

A study team member (DY or KNR) took written notes during think-alouds and exit interviews, which were deidentified and securely stored. Next, 2 investigators (DY and KNR) analyzed think-aloud and exit interview data using content analysis, informed by elements of the Technology Acceptance Model and usability theory [20,21]. The results were organized around the major overarching themes of the usefulness and usability of e-consultations for AYA care.

After reviewing the prototypes, participants also completed a web-based survey with 31 questions, including demographic questions and items adapted from the Technology Acceptance Model survey [20] and the end user computer satisfaction survey [22]. Demographic data collected in the web-based survey were analyzed with descriptive statistics using Stata 14 (StataCorp). Web-based survey responses from PCPs and adolescent medicine specialists during phases 1 and 2 were compared using the Kruskal-Wallis test to ensure that we did not overly adapt the design to favor one group (PCPs vs adolescent medicine specialists) at the expense of the needs and preferences of the other.

## Results

### Participants

Participants included 12 general pediatrician PCPs and 12 adolescent medicine specialists (Table 1). Participants included individuals in training (n=6) as well as individuals with more than 20 years of practice (n=9). Participant characteristics were similar in each phase with the exception of years of experience, with more clinicians with more than 21 years in practice in phase 1 (n=7; 6 PCPs and 1 adolescent medicine specialist) than in phase 2 (n=2; 1 PCP and 1 adolescent medicine specialist).

**Table 1:** Participant characteristics of phase 1 and phase 2.

Characteristics			Phase 1: static prototype (n=12), n (%)		Phase 2: EHR^a^ prototype (n=12), n (%)
**Clinician type**					
	PCP^b^	6 (50)		6 (50)	
	Adolescent medicine specialist	6 (50)		6 (50)	
**Duration of clinical practice**					
	Currently in training	2 (17)		4 (33)	
	0-5 years	2 (17)		4 (33)	
	6-10 years	1 (8)		0 (0)	
	11-20 years	0 (0)		2 (17)	
	>21 years	7 (58)		2 (17)	
**Clinical time (half-days weekly)**					
	0-2	3 (25)		4 (33)	
	3-4	4 (33)		3 (25)	
	5-6	3 (25)		2 (17)	
	>7	2 (17)		3 (25)	
**Gender**					
	Male	3 (25)		2 (17)	
	Female	8 (67)		10 (83)	
	Other or prefer not to answer	1 (8)		0 (0)	

^a^EHR: electronic health record.

^b^PCP: primary care physician.

### Perceived Usefulness

Regarding the usefulness of e-consultations, PCPs and adolescent medicine specialists perceived potential advantages for patients, including themes of improving access to care, increasing timeliness and convenience of care, reducing travel burden, and enhancing the role of the patient-centered medical home (Table 2).

**Table 2:** Participant perspectives on the usefulness of e-consultations.

Domain	Primary care themes	Adolescent medicine themes
Relative advantage for patients (vs traditional options)	Reduces visits (+)^a^ Increases timeliness of care (+) Increases access for patients with barriers (+) Increases communication (+) Improves health care for patients (+)	Reduces visits (+) Increases timeliness of care (+) Increases convenience (+) Decreases transportation barriers (+) Keeps care within the medical home (+) May lead to increased PCP^b^ visits in lieu of specialty visits (+/−)^c^
Relative advantage for clinicians (vs traditional options)	Facilitates communication with a specialist in a more structured way (+) Value in PCP getting answers for patient and family (+) Takes a lot of PCP time (−)^d^ Adoption will require adequate payment for time (+/−) May be better for some clinical scenarios than other (+/−)	Consult will provide structure and documentation to activities specialists already do (+) Could use to connect with other specialists or subspecialists (+) May help to ensure PCP provides information needed for a consult (+)
Value for generalists versus specialists	Makes the process easier for specialists but increases work for PCPs (−)	PCPs may not be comfortable with carrying out adolescent medicine plan (−)
Complexity	Difficult to anticipate what specialist needs to know (−)	Difficult to know what PCPs should be expected to include in a consult (−) Difficult to know what management PCPs are comfortable initiating (−)
Compatibility	Slightly repetitive of documentation for the visit itself (−)	Anticipate fitting easily into everyday workflow (+) Fitting into the workday will depend on the volume (−) Interoperability issues with other EHRs^e^ (−) Would be ideal if integrated with the scheduling process (+/−)
Learning incentive	Will lead to less reliance on specialists in the future (+)	Will help PCPs learn for future patients (+) May lead to fewer consults in the future (+)
Expected frequency	Would use on a regular basis (+)	Definitely anticipate using (+)

^a^Theme perceived as a positive effect of e-consultations.

^b^PCP: primary care physician.

^c^Theme perceived as a positive or negative effect of e-consultations.

^d^Theme perceived as a negative effect of e-consultations.

^e^EHR: electronic health record.

PCP and adolescent medicine specialist perceptions of the advantages of e-consultations for clinicians were more variable. Although adolescent medicine specialists felt that e-consultations would provide structure and documentation to activities that specialists already do, PCPs identified a burden on their time and desired adequate reimbursement in a fee-for-service environment. Similarly, regarding the value for generalists versus specialists, PCPs felt that e-consultations would make the referral process easier for specialists while increasing the workload of PCPs. Regarding the complexity of the process, adolescent medicine specialists expressed concern that PCPs may not be comfortable carrying out their management plans, and both groups noted the complexity of anticipating the knowledge that the other clinician would need. Regarding the compatibility of e-consultations, PCPs raised concerns about duplicating their work, and adolescent medicine specialists expressed varying degrees of concern about incorporating e-consultations into their daily workflow and interoperability with other EHRs. Both PCPs and adolescent medicine specialists noted learning incentives related to increasing PCP knowledge of AYA health concerns and decreasing reliance on specialists for this care in the future. Both groups anticipated the frequent use of e-consultations.

### Perceived Usability

Efficiency was a common focus for both PCPs and adolescent medicine specialists, with comments falling into four themes. First, participants recognized the importance of reducing documentation time by avoiding redundancy, minimizing free-text entry, and using drop-down menus (Table 3). Second, they recommended organizational changes to emphasize critical information, including prompting for the specific consult question at the beginning of the templated note. Third, they encouraged maximizing the use of autopopulated data fields. Fourth, they balanced these recommendations for efficient documentation with the need for adequately detailed information. For example, an adolescent medicine specialist recommended using diagnosis-specific templates tailored to referral reason, whereas a PCP worried that too many prompts might be interpreted as guidance to perform history or exam components that may be unnecessary and overly burdensome for specific patients.

Comments related to usability also frequently addressed reducing potential errors within the four themes. First, recommendations to use autopopulated fields to enhance efficiency were tempered with the acknowledgment that autopopulated fields can contain inaccurate or outdated information, leading to recommendations to follow autopopulated data fields with prompts to encourage PCP review and annotation of autopopulated data. Second, the importance of ensuring accurate information for interprofessional communication was emphasized. Third, both PCPs and adolescent medicine specialists advocated for prompts for specific patient and clinical information. These recommendations included prompts for PCPs relevant to the care of AYAs (eg, affirmed name and pronouns), prompts for adolescent medicine specialists to enhance the specificity of their recommendations (eg, exact laboratory test order number), and simple text changes to enhance clarity overall (eg, replacing *follow-up with adolescent* with *follow-up with Adolescent Medicine Clinic*). Fourth, the importance of prompts related to confidentiality was noted both to inform follow-up communication and ensure appropriate confidential documentation within the EHR, if needed.

Comments related to the affective experience of using e-consultations were mixed. Although some users found the amount of data entry to be frustrating and burdensome, others commented that the process was straightforward and clear. Both PCPs and adolescent medicine specialists felt that the e-consultation process was generally easy to learn with minimal training.

**Table 3:** Participant perspectives on the usability of e-consultations.

Domain and theme		Subtheme
**Efficiency**		
	Reduce documentation time	Avoid redundant documentation (PCP^a^ and AM^b^) Minimize free text (PCP and AM) Use drop-down menus when possible (PCP and AM) Allow the ability to incorporate images (AM)
	Organize to prioritize relevant data and questions	Place question to a specialist upfront to frame the consult (PCP and AM)
	Maximize autopopulated data field use	Pull in existing data fields (eg, family history) to avoid redundant data entry (PCP) Place free text after relevant autopopulated data to avoid duplicating information (PCP) Allow objective data to be pulled in and interpreted (eg, BMI percentiles; AM)
	Balance efficiency of documentation with the efficiency of the process	Develop diagnosis-specific templates to enhance efficient data sharing (AM) Template prompts may be interpreted as a mandate for PCP to obtain information and may increase the burden of the documentation process (PCP)
**Freedom from errors**		
	Minimize potential errors in autopopulated data in EHR^c^	Autopopulating data fields contain errors and need PCP review (AM and PCP) Data with a high error rate should be entered rather than pulled in (PCP)
	Ensure accurate information for interprofessional communication	Specialist needs information on how to get in touch with PCP if needed (PCP and AM) Communicating with PCP by phone is sometimes better than electronically (AM) PCP would like clarity about which specialist is receiving the consult (PCP)
	Maximize clarity of prompts for specific patient and clinical information	Maximize clarity of the desired outcome of consult (PCP) Provide specific prompts for key information (eg, preferred language, pronouns; AM) Maximize clarity of language (AM) Templates tailored to specific complaints may reduce missing key information (PCP) Specialist reply templates should use prompts for specific recommendations (eg, lab test order numbers, medication dosing, follow-up interval; PCP and AM)
	Maximize clarity of prompts about confidentiality and communication	Specify parent involvement (or lack thereof) in consultation (AM) Specify the degree of confidentiality to be maintained (AM and PCP) Specify whether adolescent, parent, or PCP could be contacted (AM)
**Affective experience**		
	Potential frustration versus straightforward	Amount of data entry could be frustrating and increase cognitive burden (PCP) Use blank space and text formatting to increase readability (PCP) System is straightforward and clear (AM)
**Learnability**		
	Learnable	System is easy to learn and use; building on familiar design is helpful (PCP) Recommend focused training in the use of e-consultations (<20 minutes; AM)

^a^PCP: primary care physician.

^b^AM: adolescent medicine.

^c^EHR: electronic health record.

### Survey Results

**Figure 1:**
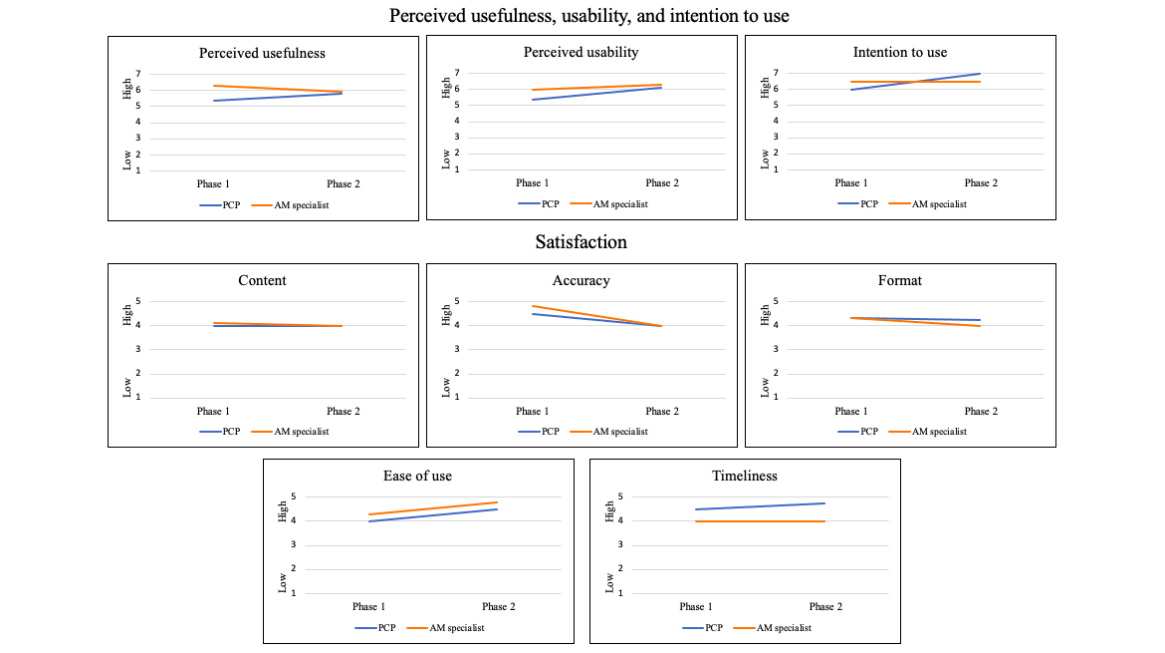
Perceived usefulness, usability, intention to use, and system satisfaction. AM: adolescent medicine; PCP: primary care physician.

During phase 1, PCPs’ responses trended lower for perceived usefulness and usability compared with adolescent medicine specialists (*P*=.05; Figure 1). During phase 2, PCPs’ perceptions of these domains increased, such that PCPs and adolescent medicine specialists had more similar assessments of e-consultations.

During the timed tasks during phase 2, the median time for PCPs to enter the mock e-consultation was 6 minutes 9 seconds (range 4 minutes 31 seconds-7 minutes 26 seconds). The median time for subspecialists to review and respond to the mock e-consultation was 7 minutes 49 seconds (range 3 minutes 56 seconds-11 minutes 8 seconds).

### Iterative Design Changes

During the first phase of development, we made changes based on feedback as participants reviewed the static prototype. For the PCP note template requesting the e-consultation, we moved the consult question for the adolescent medicine specialist to the beginning of the note template, changed the order of items to enhance accuracy and efficiency when autopopulated text was pulled in, and reduced the number of unique prompts across the review of systems, physical exam, family history, and social history fields. Although these portions were streamlined, other specific prompts relevant to the care of AYAs (eg, gender identity, confidentiality, and contact information for patients and families, and PCP) were enhanced. For the adolescent medicine specialist note template responding to the e-consultation, substantially more details were added regarding follow-up recommendations (eg, location, interval, and the specific adolescent medicine specialist to be seen if relevant).

**Figure 2:**
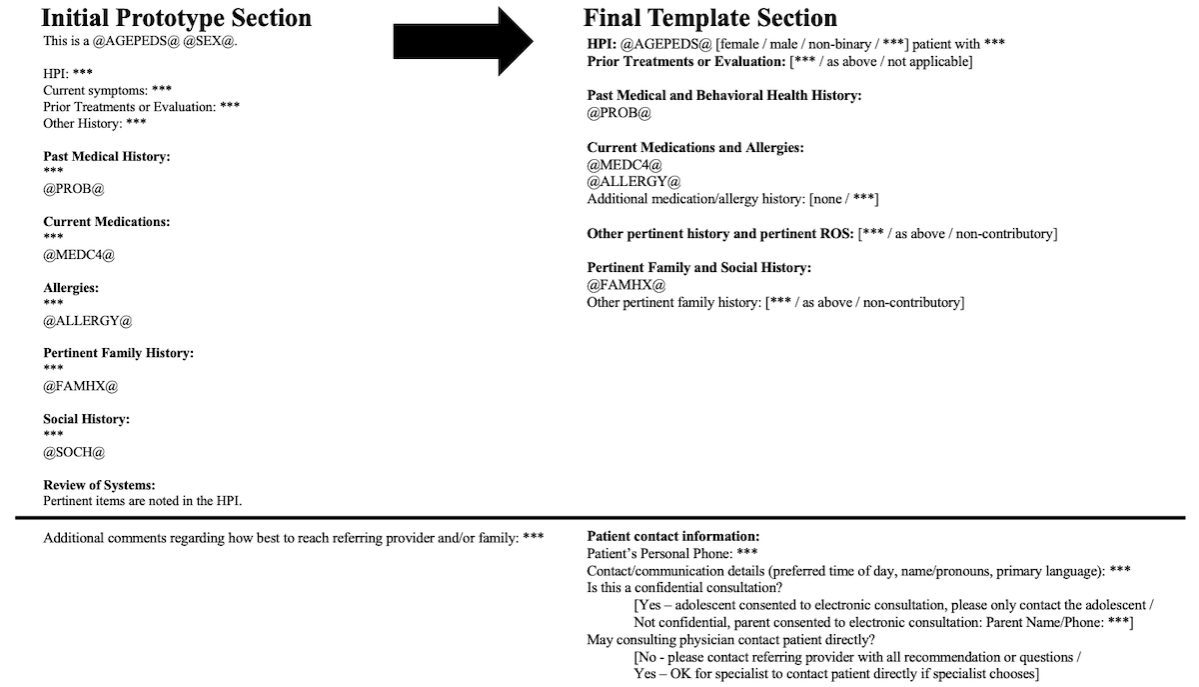
Changes in primary care physician e-consultation request note template through the iterative design process.

**Figure 3:**
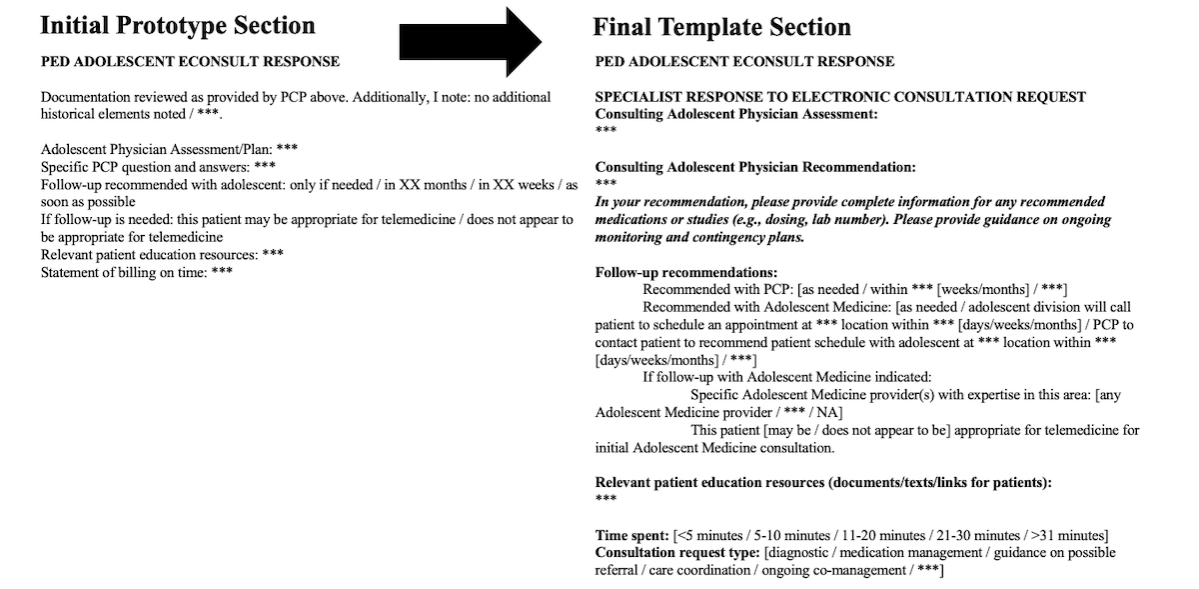
Changes in adolescent medicine specialist e-consultation response note template through the iterative design process.

During the second phase of development, we made further changes based on feedback as participants interacted with an EHR prototype (Figures 2 and 3). We added drop-down boxes to most open-ended prompts to allow rapid selection of *as above* if a clinician had already addressed a specific prompt. We consolidated multiple prompts for review of autopopulated text throughout the template into one prompt, which was placed after autopopulated information on past medical history, allergies, and medications, providing a single opportunity to review and add to these automated fields. In addition, prompts were added to pull the objective data with interpretation (eg, BMI percentiles). Furthermore, we clarified the language around contact information and confidentiality expectations for the PCP, adolescent medicine specialist, and patient and included free-text response options to clarify complex privacy concerns if needed (eg, providers may communicate with one parent but not the other). The final note templates are included in their entirety in Multimedia Appendix 1.

## Discussion

### Principal Findings

We used a replicable user-centered design process to develop and refine e-consultation templates and processes for general pediatrician consultation with adolescent medicine specialists. We qualitatively analyzed user feedback during two phases of development to guide the revision of our e-consultations and produced a final prototype with similar perceived usefulness and usability among PCPs and adolescent medicine specialists. Our iterative design process and feedback themes may inform the development of similar e-consultation systems in other settings. In particular, by engaging experts in pediatric and adolescent health in our user-centered design process, we identified specific considerations for the use of e-consultations with AYAs to promote safe, equitable, and high-quality care for this population.

Participants identified both opportunities and risks for the use of e-consultations with AYAs related to confidentiality. A major perceived advantage was the potential to enhance access to confidential care for AYAs. AYAs may delay or forgo care because of a lack of knowledge of how or where to obtain services, transportation challenges, and concerns about maintaining confidentiality [23,24]. e-Consultations were perceived as a means to ameliorate these barriers faced by AYAs in traditional primary care to subspecialty referral models by allowing adolescent medicine specialist-guided care of confidential health concerns either without the need for an in-person adolescent medicine visit or with PCP-facilitated handoff to adolescent medicine care. By providing point-of-care education for PCPs during e-consultations, this process may also enhance PCP’s ability to deliver care for future confidential health concerns. A perceived threat to confidentiality was the potential for miscommunication between PCPs and adolescent medicine specialists regarding patients’ preferences for contact and expectations of confidentiality. To reduce this risk, it was recommended that e-consultations for use with AYAs include prompts to document patients’ preferred contact information and explicit expectations surrounding confidentiality. Such prompts could have the additional benefit of encouraging PCPs to discuss laws governing confidential health care for minors and reserve time for private conversations with AYAs during their visits, features of pediatric primary care visits associated with increased disclosure of health concerns that may otherwise be lacking [25]. As laws regarding adolescent confidentiality and consent may be complex and vary among states, those developing e-consultations for AYAs may consider including details of state-specific adolescent privacy regulations to assist PCPs who are unfamiliar. Another perceived threat to confidentiality was unintended caregiver proxy access to e-consultation documentation within the EHR or receipt of an explanation of benefit statements or copays, all of which are previously identified barriers to confidential care for AYAs in general [26,27]. To reduce this risk, those developing e-consultations for use with AYAs should consider local system capacity to restrict the sharing of EHR documentation through patient portals and assess the potential to reduce breach of confidentiality through insurer explanation of benefit statements and copayments. In addition, both PCPs and adolescent medicine specialists who use e-consultations with AYAs should discuss these risks with their AYA patients and examine local options for limiting EHR documentation sharing with caregiver proxies, such as through confidential note types.

Participants further identified opportunities for the use of e-consultations with AYAs to facilitate communication. Overall, participants perceived e-consultations as a beneficial way to enhance communication between clinicians and AYAs and between PCPs and specialists. Both PCPs and adolescent medicine specialists suggested that e-consultations could increase centralized communication from the PCP to their patients, which was seen as promoting high-quality and coordinated care within the patient-centered medical home. e-Consultations were further seen as benefiting AYA health care by facilitating immediate and ongoing communication between PCPs and adolescent medicine specialists in a way not achieved through traditional referral methods [28,29]. The immediate discourse opened through e-consultations was seen as enhancing care by avoiding unnecessary visits to adolescent medicine specialists, identifying when alternative visit types (eg, telemedicine visits) might be appropriate, and triaging the immediacy with which patients should be seen. The ongoing communication facilitated by e-consultations was perceived to both enhance coordination of care and provide valuable opportunities for PCPs to enhance their adolescent health skills, a finding suggested in prior qualitative work and hinted at through a study of referral patterns throughout time [30,31].

AYA-specific e-consultation templates were also seen as a means of enhancing the quality of care for AYAs. Perceived opportunities to improve AYA care delivered by PCPs included placing prompts in PCP templates for patients’ names and pronouns—information integration to the sensitive and respectful care of AYAs that may otherwise not be elicited, may not align with EHR documentation, and which AYAs may be reticent to disclose without such signals from providers that they are in a safe space [32]. Condition-specific templates were also identified as an opportunity to ensure that necessary clinical information is transmitted from PCPs to adolescent medicine specialists to allow accurate, timely, and informed recommendations. Although participants saw potential in the ability to specify the information needed for effective collaboration through e-consultation templates, they also acknowledged that excessive documentation might become burdensome and decrease the uptake of e-consultations, a concern raised in prior studies as well [19].

The ability to refine the minimum required documentation to balance efficiency and precision in e-consultations shows the value of iterative user-centered design in this context. Using a multiphase development process including think-alouds, exit interviews, and surveys with representative users, we elicited increasingly specific feedback that facilitated the first broad updates to the content and layout of the e-consultation prototypes and later finer changes to the template language. By engaging users who were both generalists and specialists, we were able to increase the alignment of PCP and adolescent medicine specialist perceptions throughout time through iterative modification with input from both sides. Such user-centered design has the potential to increase the uptake and acceptability of new health information technologies [33] and may be valuable in tailoring existing technologies to promote optimal care of populations with unique health care needs, including AYAs.

### Limitations

The user-centered design process outlined here is both a strength and a limitation of this study. Our study included a small number of participants from a single geographic area and practice within a specific clinical context. This allowed the creation of a tailored e-consultation system that may be readily implemented within our local health system but which may lack generalizability or acceptability for other health systems broadly, including those without compatible EHR systems across general pediatric and adolescent medicine specialty practices (although the lessons learned about needed content in these notes could apply to other systems as well). As a result, the themes identified from this development process should be viewed as starting places for conversations in systems other than final recommendations. We also note that the years in practice for PCPs and adolescent medicine specialists varied from phase 1 to phase 2. As this analysis focused on design rather than the implementation of e-consultations, it does not include the evaluation of quality or outcomes of use. Although e-consultations were perceived to have high usefulness and usability during the design process, new concerns may arise during implementation [34,35]. In addition, this study was conducted before dramatic increases in the use of live-interactive telemedicine during the COVID-19 pandemic [8], which may alter the perceived usefulness and intention to use e-consultations in a shifting pediatric care delivery landscape. Finally, we did not include AYAs or caregivers in the design process. Both groups may have additional preferences regarding system design, as evidenced by work with pediatric patients and caregivers [36,37], such that additional work specifically with AYAs and AYA caregivers is warranted.

### Conclusions

In conclusion, we used an iterative user-centered design process to develop and refine e-consultations for use by general pediatric PCPs and adolescent medicine specialists. We used qualitative analysis of user feedback elicited during the design process to identify themes relevant to the development and implementation of similar e-consultation systems for use in other health systems or among other patient populations. By engaging experts in the care of children and adolescents in the design process, we identified both opportunities and risks related to confidentiality, communication, and the use of tailored documentation prompts that should be considered in the development and use of e-consultations with AYAs.

## Appendix

Multimedia Appendix 1 Final templates for adolescent medicine e-consultation requests and responses.

## Abbreviations

AYA: adolescent and young adult
EHR: electronic health record
PCP: primary care physician
