# Supplementary material for: Optimizing e-Consultations to Adolescent Medicine Specialists: Qualitative Synthesis of Feedback From User-Centered Design
Source: JMIR Hum Factors. 2021 Aug 5;8(3):e25568. doi: 10.2196/25568 (PMC8380586; doi:10.2196/25568)
Supplement: Multimedia Appendix 1 [file humanfactors_v8i3e25568_app1.docx]

**Outpatient Electronic Consultation Request to Adolescent Medicine Specialist**

**Question for Specialist:**

***

**HPI:** @AGEPEDS@ {female / male / non-binary / ***} patient with ***

**Prior treatments or evaluation:** {*** / as above / not applicable}

**Past Medical and Behavioral Health History:**

@PROB@

**Current Medications and Allergies:**

@MEDC4@

@ALLERGY@

Additional medication/allergy history: {none / ***}

**Other pertinent history and pertinent ROS:** {*** / as above / non-contributory}

**Pertinent Family and Social History:**

.famhx

Other pertinent family history: {***/ as above / non-contributory}

**Objective:**

**Date of last visit:** .lastencthisdept

@VITALSM@

.bmi

.bpfa

**Pertinent exam *(if relevant, please include abdominal/GU/tanner stage/psych):* *****

**Pertinent laboratory or radiologic studies done to date:**

***

**Referring provider’s working diagnosis, evaluation, treatment, and question details:**

***

**Discussion with patient/family to date:**

{*** / as above}

**Patient contact information:**

Patient’s Personal Phone: ***

Contact/communication details (preferred time of day, preferred name/pronouns, primary

language): ***

Is this a confidential consultation?

{Yes – adolescent consented to electronic consultation, please only contact the adolescent / Not confidential, parent consented to electronic consultation: Parent Name/Phone ***}

May consulting physician contact patient directly?

{No - please contact referring provider with all recommendations or questions

/ Yes - OK for specialist to contact patient directly if specialist chooses}

**Referring provider's direct contact number if specialist needs to contact:** ***

**Referring provider time spent preparing electronic consultation:** {<5 minutes / 5-10 minutes / 11-20 minutes / 21-30 minutes / >31 minutes)

**Consultation request type:** {diagnostic / medication management / guidance on possible referral / care coordination / ongoing co-management / ***}

**The Adolescent Medicine provider will respond within 72 business hours with recommendations for you to relay to your patient. For requests that need more urgent response, please page the Adolescent Medicine attending on call.**

|  |
| --- |

**Adolescent Medicine Specialist Response to Electronic Consultation**

**Consulting Adolescent Physician Assessment:**

***

**Consulting Adolescent Physician Recommendation:**

***

***In your recommendation, please provide complete information for any recommended medications or studies (e.g., dosing, lab number). Please provide guidance on ongoing monitoring and contingency plans.***

**Follow-up recommendations:**

Recommended with PCP:

{as needed / within *** {weeks/months} / ***}

Recommended with Adolescent Medicine:

{as needed

/ adolescent division will call patient to schedule an appointment at *** location within *** {days/weeks/months}

/ PCP to contact patient to recommend patient schedule with adolescent at *** location within *** {days/weeks/months} / ***}

If follow-up with Adolescent Medicine indicated:

Specific Adolescent Medicine provider(s) with expertise in this area:

{any Adolescent Medicine provider / *** / NA)

This patient {may be / does not appear to be} appropriate for telemedicine for initial Adolescent Medicine consultation.

**Relevant patient education resources (documents/texts/links for patients):**

***

**Time spent:** {<5 minutes / 5-10 minutes / 11-20 minutes / 21-30 minutes / >31 minutes)

**Consultation request type:** {diagnostic / medication management / guidance on possible referral / care coordination / ongoing co-management / ***}

|  |
| --- |

Templates include spaces for users to enter free-text responses (***) and choose drop-down menu selections (response options contained in braces {} and separated by slashes [/]). Templates auto-populate information from the electronic health record in the spaces indicated by smart phrases or dot phrases (eg, @AGEPEDS@ and .famhx).
